# Exploring the unmet needs of family planning: Insights from a cross-sectional study in a rural area of coastal Karnataka, India

**DOI:** 10.1177/22799036251397747

**Published:** 2026-01-20

**Authors:** Ratna Jay, Yash Alok, Manjula A., Ashwini Kumar, Sanjay Kini B.

**Affiliations:** 1 Department of Community Medicine, Kasturba Medical College, Manipal, Manipal Academy of Higher Education, Manipal, Karnataka, India

**Keywords:** family planning, unmet needs, contraception, Coastal Karnataka, reproductive health

## Abstract

**Objectives:**

Despite decades of programmatic initiatives, family planning remains a public health concern in India. The present study was conducted to evaluate the prevalence and determinants of unmet needs of family planning in a rural area of Udupi district in Coastal Karnataka, South India.

**Methods:**

A cross-sectional study was conducted among 565 married women aged 18–49 years, using a semi-structured questionnaire. Information on socio-demographic factors, contraceptive awareness, and contraceptive use was collected. Data was analysed to find the prevalence of unmet need for family planning and to identify associated factors.

**Results:**

A total of 565 married women aged 18–49 years participated. The overall prevalence of unmet need for family planning was 40.3% (13.5% for spacing and 26.9% for limiting). The prevalence rate of contraception was determined to be 46%, with 260 women currently using contraceptives, 77 past users, and 228 never users. Multivariate analysis showed that age below 30 years (AOR = 0.54, 95% CI: 0.30–0.98, *p* = 0.043), place of last delivery at home (AOR = 2.94, 95% CI: 1.04–8.36, *p* = 0.042), ideal number of female children as one (AOR = 4.82, 95% CI: 1.00–18.87, *p* = 0.050), wanting more than two children at marriage (AOR = 2.46, 95% CI: 1.09–5.57, *p* = 0.030), and lack of awareness about IUCD (AOR = 0.15, 95% CI: 0.03–0.71, *p* = 0.017) were significant independent determinants of unmet need for family planning.

**Conclusion:**

These results demonstrate the significant discrepancy between knowledge and use of contraceptive methods in this area with otherwise positive health indicators, indicating the need for focussed interventions that address sociocultural barriers and misconceptions to increase the accessibility and acceptance of family planning services.

## Introduction

### Background

In 1952, the Indian Government established a National Programme for Family Planning (NPFP) to limit the population growth.^1^ Over the decades, the programme has been radically transformed and implemented under a larger and more extensive programme of RMNCAH + N (Reproductive, Maternal, Newborn, Child Health, and Adolescents Health + Nutrition) Programme to include the closely related topics of reproductive health and maternal/infant mortality and morbidity.^2^

Creating awareness of contraception and reproductive health is an important goal for RMNCH + A. However, it has been documented through various studies^3,4^ that despite the best efforts of the programme, the accessibility and awareness around contraception have been lacking, especially among sexually active women, and even among those who respond negatively to desiring pregnancy shortly or those who want to delay the subsequent childbirth. There is a divergence between the reproductive intentions and the use of contraception in this segment of the population. This gap is referred to as Unmet need.^5^

Unmet need is an indicator to measure how the health system and the social conditions support a woman’s reproductive intentions to delay or limit childbirths.^5^ It is also a barometer to assess the success of country’s reproductive health programmes. The variable of unmet needs is an assessment metric to indicate the additional degree of need to delay or limit pregnancies, which is a supplement to the contraceptive prevalence rate. Overall Contraceptive Prevalence Rate (CPR) has increased substantially in India from 53.5% in 2015–16 to 67% in 2019–2020 at national level.^6^ It has been observed that there is a significant unmet need for contraception in India as per the District Level Health Survey-3 (DLHS-3).^7^ The survey reported that the total unmet need for family planning in India is 21.3 %, with 7.9% unmet need for spacing and 13.4% unmet need for limiting.^7^ As per the National Family and Health Survey, there has been a noteworthy decline in the Unmet need for family planning, wherein the figures have decreased from 12.9% (NFHS-4) to 9.4% (NFHS-5).^6,8^

The world’s population currently is 7.7 billion, and it is estimated to increase to 9.7 billion by the year 2050. India is the most populated country with a population of 1.39 billion, representing 17.76% of the world’s population.^9^ The unmet need for family planning and unplanned pregnancy are important public health concerns for India in this context. Studies have documented the overall effect of an unintended pregnancy on maternal depression and parenting stress.^10,11^ Additionally, unplanned pregnancies resulted in adverse outcomes of pregnancies like low birth weight and pre-term newborns.^12^ Studies have reported that, of all the Sustainable Development Goals (SDGs), Family Planning has the second best ‘return-on-investment (after education)’.^13^ The Sustainable Development Goal (SDG) indicator 3.7.1, which is the ‘Proportion of women of reproductive age group who have their need for family planning satisfied with modern methods’ informs the global review of SDG target 3.7.^14^ Unmet need for family planning in Udupi district remains above the Karnataka state average, despite the district’s favourable demographic indicators.^6,8^ Udupi has a low total fertility rate (TFR) of approximately 1.2, which is below the replacement level and comparable to developed countries. The district also boasts a high literacy rate exceeding 85%, indicating strong educational attainment. This paradox of persistent unmet need within a context of low fertility and high literacy highlights the unique socio-cultural and health system factors that warrant focussed investigation in Udupi. Hence this study was conducted to estimate the prevalence and to evaluate the factors associated with unmet need for family planning in a rural area of Udupi district of Karnataka state in South India.

## Materials and methodology

This was a cross-sectional study conducted in a rural area of the Udupi district of Karnataka state in South India. A rural area was chosen as the unmet need for family planning is higher in rural compared to urban populations, as evidenced by NFHS-5 (2019–21), where the total unmet need was 9.9% in rural areas versus 8.4% in urban areas, and unmet need for spacing was 4.3% in rural versus 3.6% in urban areas. Conducting the study in the institution’s rural field practice area also ensured the feasibility of data collection and close engagement with the community. Married women in the age group 18–49 years, living in the households of 7 selected sub-centre areas of Udupi taluk were included. Women who were separated/ widowed/ divorced, and who had undergone hysterectomy were excluded from the study. The sample size for the present study was obtained using the formula *n* = $\frac{z^{2}pq}{d^{2}}$ . According to a previous study conducted in a rural area of Tamil Nadu, by Prasad et al.,^15^ the prevalence of unmet need for family planning was found to be 31%. Considering this prevalence, for a precision of 4%, α error of 5%, and a non-response rate of 10%, we obtained a sample size of 564. Convenience sampling with proportional allocation was used to obtain the desired sample size. The number of participants drawn from each of the seven selected sub centres areas corresponded to the proportion of the eligible couples in that area. A detailed table with the names of the sub-centres and the number of participants from each is provided in the Supplementary File S1.

The study duration was from May 2022 to June 2024, with data collection period from 9th March 2023 to 7th December 2023. The participant information sheet was used to describe the objectives and the purpose of the study in the local language. Written informed consent was obtained from the participants before they were recruited for the study.

A pretested, semi-structured questionnaire (Supplemental File S2) was administered to all study participants through personal interviews during the house visits. Subjects fulfilling the inclusion criteria were interviewed using the questionnaire which had the following sections:

a. Socio-demographic information which included age, education, and occupation, Socio-economic status which was calculated using the modified Uday Pareek Scale.^16^
 which is widely applied in rural settings. This scale considers multiple components, including family income, occupation, education, caste, social participation, landholding, and housing, to generate a composite SES score.
b. Reproductive history of participants which included age at marriage, total number of pregnancies, live births, abortions, number of male children and number of female children.
c. Knowledge and attitude towards family planning which included questions related to the ideal age of women to get married, ideal age to have first child, ideal number of children, ideal age spacing between children, perception at the time of marriage regarding the ideal time to have the first child.
d. Information regarding use of contraception: Participants were classified as Current users, former users, non-users based on whether they were using any of the modern contraceptive methods during the previous fertile period of their menstrual cycle. Information regarding method of contraception used currently, duration of use and satisfaction with the current method was also noted.
e. Emergency contraception usage and abortion-related information was also gathered.
f. Information regarding sexually transmitted infections or reproductive tract infections suffered by the participants.

### Operationalisation of study variables

○ Age of the participant: Recorded in completed years and categorised as <20, 21–30, 31–40, and >41 years.
○ Age at marriage: ≤21 years and >21 years.
○ Age at birth of first child: ≤20 years, 21–30 years, and >30 years.
○ Total number of pregnancies: 1, 2, or ≥3.
○ Total number of live births: 1, 2, or ≥3.
○ Total number of abortions: none, 1, or ≥2.
○ Number of children alive: 1, 2, or ≥3.
○ Number of male children alive: none, 1, or ≥2.
○ Number of female children alive: none, 1, or ≥2.
○ Place of delivery of the last child: Government facility or home/private facility.
○ Religion: Hindu, Muslim, or Christian.
○ Education: Primary, secondary, pre-university college, or graduate and above.
○ Occupation: Homemaker/student/unemployed; unskilled/semi-skilled/skilled

### Operational definitions

i. Unmet need for modern family planning methods: The proportion of women of reproductive age who are not using any modern contraceptive methods but wish to delay their next pregnancy (unmet need for spacing) or prefer not to have any more children (unmet need for limiting). The combined total of unmet need for limiting and spacing represents the overall unmet need for family planning.^4^
ii. Modern spacing methods include contraceptive pills, condoms, injectable contraceptives, intrauterine contraceptive devices (IUCD), and emergency contraception. Modern limiting methods include male and female sterilisation.^4^
For analysis, the outcome variable, unmet need for family planning, was operationalised as a binary variable. Unmet need present referred to women who either wished to delay their next pregnancy (unmet need for spacing) or did not want any more children (unmet need for limiting) but were not using any modern contraceptive method. Unmet need absent referred to women who were either using modern contraceptive methods or were planning to have a child and therefore not using modern contraception.

**Figure 1.**
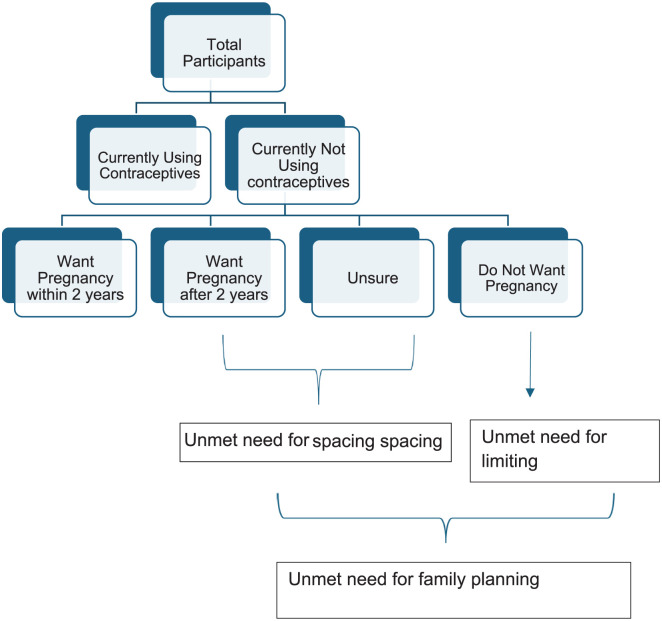
Flowchart depicting the unmet need for family planning.

Figure 1 depicts the operational definitions considered for unmet need of family planning for both spacing and limiting.

### Data analysis

Data was analysed using Statistical Package for Social Sciences (SPSS) version 15. Descriptive statistics were employed to characterise the study population, with categorical variables expressed as frequencies and percentages. Continuous variables were recorded but were categorised into groups for analysis, hence presented as categorical variables in the results.

For each categorical independent variable, the overall p value was calculated using univariate logistic regression. Variables with overall *p* value < 0.20 in univariate analysis were considered for inclusion in the multivariate logistic regression model. Multivariate logistic regression analysis was conducted to identify independent predictors of unmet need for family planning while controlling for potential confounders. A *p*-value less than 0.05 was considered statistically significant for all analyses.

## Results

We enrolled 565 participants in our study. Unmet need was significantly higher among Muslim women (46.4%) compared to Hindus (35.0%; OR = 1.6, 95% CI: 1.1–2.3, *p* = 0.009). Table 1 depicts the association of sociodemographic characteristics, including age, religion, education, occupation, and socioeconomic status, with unmet need for family planning among the study participants.

**Table 1.** Association of sociodemographic characteristics with the unmet need for family planning among the study participants (*n* = 565).

Characteristic		Unmet need present	Unmet need absent	*p* value		Unadjusted OR (95% CI)
		Frequency (%)	Frequency (%)			
Age of the participant in years	≤30	189 (33.5)	89 (47.1)	0.815	0.013	1.571 (1.102–2.239)
	>31	376 (66.5)	136 (36.2)	0.173		1
Religion	Hindu	116 (35.0)	215 (65.0)	Ref	0.022	1
	Christian	13 (48.1)	14 (51.9)	0.177		1.721 (0.783–3.784)
	Muslim	96 (46.4)	111 (53.6)	0.009		1.603 (1.125–2.285)
Education	Primary	39 (31.2)	86 (68.8)	0.044	0.094	0.584 (0.346–0.987)
	Secondary	82 (39.4)	126 (60.6)	0.450		0.839 (0.531–1.324)
	Pre-university college	52 (46)	61 (54)	0.722		1.098 (0.654–1.843)
	Graduate and above	52 (43.7)	67 (56.3)	Ref		1
Occupation	Housewife, student, unemployed	210 (39.8)	318 (60.2)	0.186	0.202	0.528 (0.205–1.360)
	Unskilled, semi-skilled, skilled worker	5 (26.3)	14 (73.7)	0.075		0.286 (0.072–1.137)
	Professional	10 (55.6)	8 (44.4)	Ref		1
Socioeconomic status	Upper class	23 (41.8)	32 (58.2)	Ref	0.210	1
	Upper middle class	96 (36.6)	166 (63.4)	0.472		0.80 (0.445–1.454)
	Middle class	97 (43.7)	125 (56.3)	0.802		1.080 (0.594–1.963)
	Lower middle class	9 (34.6)	17 (65.4)	0.536		0.737 (0.279–1.942)

Ref: reference category.

*p* values are overall values obtained using univariate logistic regression. Variables with *p* < 0.20 were considered for inclusion in multivariate logistic regression analysis.

**Figure 2.**
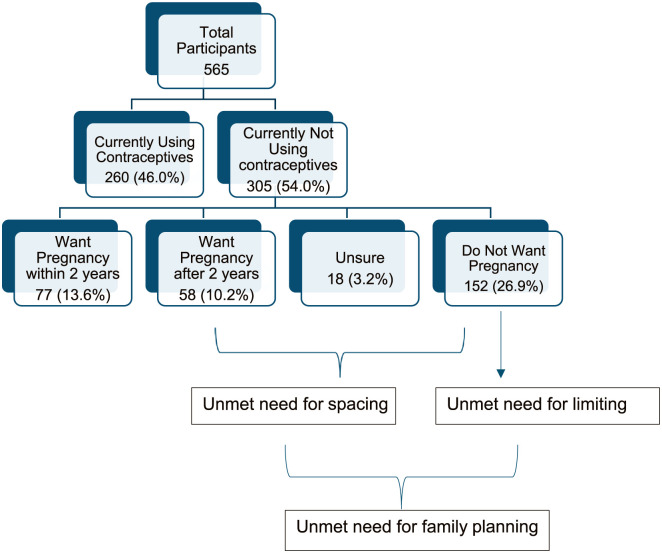
Prevalence of unmet need for family planning (*n* = 565).

A total of 565 women participated in the study. The overall prevalence of unmet need for family planning was 40.3%, of which 13.5% was for spacing and 26.9% for limiting. The contraceptive prevalence rate was 46%, with 260 women being current users, 77 former users, and 228 never users (Figure 2).

On univariate analysis it was observed that the odds of having an unmet need for family planning were 3.914, 3.121, and 3.111 times higher among women who had a single pregnancy, two pregnancies, and three or more pregnancies than women who had no pregnancies (Table 2).

**Table 2.** Association of reproductive history with unmet need for family planning (*n* = 565).

Characteristic		Unmet Need Present	Unmet Need Absent	p value		Unadjusted OR (95% CI)
		Frequency (%)	Frequency (%)			
Age at marriage (years)	<21	96 (38.2)	155 (61.8)	0.494	0.494	0.888 (0.632–1.247)
	>21	129 (41.1)	185 (58.9)	Ref		1
Age at birth of first child	<20	28 (41.4)	72 (58.6)	0.013	0.008	0.367 (0.166–0.812)
	21–30	173 (43.9)	22 (56.1)	0.392		0.739 (0.370–1.477)
	>30	18 (14.5)	17 (20.5)	Ref		1
Total number of pregnancies	0	6 (13.5)	28 (20.5)	Ref	0.049	1
	1	52 (45.6)	62 (68.6)	0.005		3.914 (1.505–10.178)
	2	101 (100.4)	151 (59.9)	0.015		3.121 (1.248–7.809)
	≥3	66 (40)	99 (60)	0.017		3.111 (1.221–7.926)
Total number of live births	0	7 (16.7)	35 (83.3)	Ref	0.008	1
	1	69 (46.9)	78 (53.1)	0.001		4.921 (1.943–12.464
	2	110 (39.7)	167 (60.3)	0.007		3.494 (1.417–8.617)
	≥3	39 (39.4)	60 (60.6)	0.005		3.877 (1.494–10.059)
Total number of abortions	0	180 (39.6)	275 (60.4)	Ref	0.623	
	1	37 (43.5)	48 (56.5)	0.494		1.178 (0.737–1.881)
	2	7 (36.8)	12 (63.2)	0.812		0.891 (0.344–2.306)
	>3	1 (16.7)	5 (83.3)	0.281		0.306 (0.35–2.637)
Total number of children alive	0	6 (15.4)	33 (84.6)	Ref	0.016	1
	1	68 (45.3)	82 (54.7)	0.001		4.561 (1.804–11.529)
	2	113 (40.1)	169 (59.9)	0.005		3.678 (1.492–9.062)
	≥3	38 (40.4)	56 (59.6)	0.007		3.732 (1.425–9.771)
Total number of male children alive	0	68 (37)	116 (63)	Ref	0.442	1
	1	107 (43.7)	138 (56.3)	0.162		1.323 (0.894–1.957)
	2	45 (36.6)	78 (63.4)	0.947		0.984 (0.613–1.580)
	3	5 (38.5))	8 (61.5)	0.914		1.066 (0.335–3.390)
Total number of female children alive	0	79 (37.3)	133 (62.7)	Ref	0.032	1
	1	94 (38.4)	151 (61.6)	0.808		1.048 (0.717–1.531)
	2	48 (52.7)	43 (47.3)	0.013		1.879 (1.143–3.089)
	≥3	4 (23.5)	13 (76.5)	0.264		0.518 (0.163–1.644)
Place of delivery of the last child	Government health facility	62 (30.1)	144 (69.9)	0.000	0.000	0.447 (0.309–0.648)
	Home	2 (50)	2 (50)	0.970		1.039 (0.145–7.466)
	Private facility	155 (49.1)	161 (50.9)	Ref		1

Ref: reference category.

p values are overall values obtained using univariate logistic regression. Variables with p < 0.20 were considered for inclusion in multivariate logistic regression analysis.

Table 3 shows the association of the perception of family planning with the unmet need for family planning. Women who perceived the ideal family size as more than two children had 1.66 times higher odds of unmet need for family planning, while those who perceived the ideal as one child had 2.13 times higher odds, compared to women who considered two children as ideal.

**Table 3.** Association of perception of family planning with the unmet need for family planning (*n* = 565).

Characteristic		Unmet need present	Unmet need absent	*p* value		Unadjusted OR (95% CI)
		Frequency (%)	Frequency (%)			
Ideal age for a woman to get married (years)	>30	1 (20)	4 (80)	0.239	0.982	0.265 (0.029–2.420)
	18–21	36 (32.43)	75 (67.5)	0.010		0.523 (0.319–0.858)
	22–25	124 (44.7)	153 (55.2)	0.437		0.859 (0.586–1.259)
	26–30	83 (48.2)	89 (51.7)	Ref		1
Ideal age to have first child (years)	Soon after marriage	104 (47.3)	116 (52.7)	1	0.327	1,447,877,956
	1–2 years after marriage	104 (46)	122 (54)	1		732,106,494
	>2 years after marriage	34 (31.2)	75 (68.8)	Ref		1
	other	3 (33.3)	6 (66.7)	1		1,376,670,843
Ideal number of children	1	11 (55)	9 (45)	0.100	0.018	2.133 (0.865–5.262)
	2	153 (36.4)	267 (63.6)	Ref		1
	>2	61 (48.8)	64 (51.2)	0.013		1.663 (1.111–2.489)
Ideal number of male children	No sex differentiation	106 (96.4)	136 (56.2)	Ref	0.397	1
	1	100 (36.5)	174 (63.5)	0.091		0.737 (0.518–1.050)
	≥2	19 (38.5)	29.5 (61.5)	1		2072
Ideal number of female children	No sex differentiation	103 (43.8)	132 (56.2)	Ref	0.039	1
	1	87 (33.6)	172 (66.4)	0.977		1.030 (0.137–7.748)
	2	33 (49.3)	34 (50.7)	0.432		0.804 (0.467–1.385)
	>2	2 (50.0)	2 (50.0)	0.019		0.521 (0.302–0.898
Ideal age gap between 2 children	Don’t know	3 (60)	2 (40.0)	0.445	0.663	2.034 (0.329–12.560)
	1 year	1 (14.3)	6 (85.7)	0.174		0.226 (0.026–1.928)
	1–2 years	8 (34.8)	15 (65.2)	0.491		0.723 (0.288–1.818)
	3–5 years	108 (40.0)	162 (60.0)	0.634		0.904 (0.597–1.369)
	>5	46 (38.0)	75 (62.0)	0.468		0.832 (0.505–1.368)
	2–3 years	59 (42.4)	80 57.6)	Ref		1
Number of children the participant planned to have	Never thought of it	193 (41.5)	272 (58.5)	0.009	0.025	2.027 (1.193–3.445)
	One	7 (58.3)	5 (41.7)	0.030		4 (1.145–13.970)
	Two	21 (25.9)	60 (74.1)	Ref		1
	More than two	4 (57.1)	3 (42.9)	0.097		3.810 (0.787–18.445)
Number of male children the participant wished to have	Never thought of it	212 (41.7)	297 (58.3)	Ref	0.994	-
	One	11 (23.9)	35 (76.1)	0.913		1.1 (0.199–6.090)
	Two	2 (22.2)	7 (77.8)			1
	More than two	0	1 (100)	1		0
Number of female children the participant wished to have	Never thought of it	213 (41.4)	301 (58.6)	0.940	0.994	0.944 (0.209–4.259)
	One	9 (20.9)	34 (79.1)	0.221		0.353 (0.67–1.870)
	Two	3 (42.9)	4 (57.1)	Ref		1
	More than two	0	1 (100.0)	1		0
Duration after marriage when the participant wanted her first child	Never thought of it	193 (41.2)	276 (58.8)	0.458	0.364	0.333 (0.018–6.063)
	Just after marriage	7 (25.0)	21 (75.0)	0.801		0.699 (0.043–11.248)
	1–2 years after marriage	18 (40.9)	26 (59.1)	0.511		0.375 (0.20–6.997)
	>2 years	6 (27.3)	16 (72.7)	Ref		1

Ref: reference category.

*p* values are overall values obtained using univariate logistic regression. Variables with *p* < 0.20 were considered for inclusion in multivariate logistic regression analysis.

Multivariate analysis showed that women aged 30 years or younger had significantly lower odds of unmet need compared to older women (AOR = 0.54; 95% CI: 0.30–0.98; *p* = 0.043). Deliveries at government health facilities were associated with reduced unmet need versus private facilities (AOR = 0.56; 95% CI: 0.33–0.97; *p* = 0.039). Women who preferred two female children had lower odds of unmet need (AOR = 0.11; 95% CI: 0.01–0.91; *p* = 0.040), whereas those desiring more than two children at marriage had substantially higher odds (AOR = 26.34; 95% CI: 5.21–133.17; *p* < 0.001). Awareness of IUCD was linked to lower unmet need (AOR = 0.15; 95% CI: 0.03–0.71; *p* = 0.017). These findings emphasise the importance of age, delivery setting, fertility preferences, and contraceptive awareness in addressing unmet family planning needs (Table 4).

**Table 4.** Association of characteristics with unmet need for family planning on Multivariate logistic regression analysis (*n* = 565).

Characteristic		Unmet need present	Unmet need absent	*p* value	Adjusted OR with 95% CI
		Frequency (%)	Frequency (%)		
Age of the participant (years)	≤30	89 (47.1)	100 (52.9)	**0.043**	0.537 (0.295–0.980)
	>30	136 (36.2)	240 (63.8)	-	1
Place of delivery of the last child	Government health facility	62 (30.1)	144 (69.9)	**0.039**	0.562 (0.326–0.971)
	Home	2 (50)	2 (50)	0.587	2.041 (0.155–26 838)
	Private facility	155 (49.1)	161 (50.9)	Ref	1
Ideal number of female children	1	87 (33.6)	172 (66.4)	0.124	0.103 (0.006–1.874)
	2	33 (49.3)	34 (50.7)	**0.040**	0.108 (0.013–0.906)
	>2	2 (50)	2 (50)	**0.001**	0.135 (0.041–0.446)
	No sex differentiation	103 (43.8)	132 (56.2)	Ref	1
Number of children the participant planned to have	Never thought of it	193 (41.5)	272 (58.5)	0.257	4.137 (0.355–48.173)
	One	7 (58.3)	5 (41.7)	**0.001**	4.025 (1.777–9.120)
	Two	21 (25.9)	60 (74.1)	Ref	1
	More than two	4 (57.1)	3 (42.9)	**0.000**	26.336 (5.208–133.172)
Awareness about IUCD	Yes	220 (40.9)	318 (59.1)	Ref	1
	No	5 (11.7)	22 (15.3)	**0.017**	0.148 (0.031–0.713)

Bolded *p*-values indicate statistically significant results (*p* < 0.05), demonstrating a significant association between the variable and unmet need for family planning.

**Figure 3.**
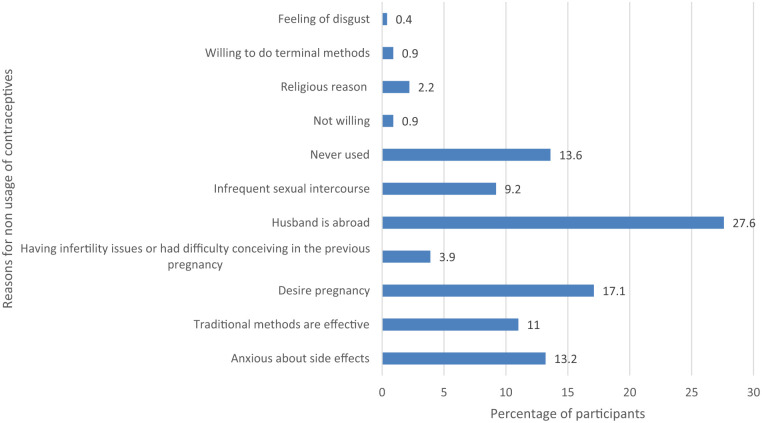
Reasons for non-usage of contraceptives among never users of contraception (*n* = 228).

Among never-users, the most frequently cited reasons for not using contraception were husband living abroad (27.3%), fear of side effects, and lack of perceived need (Figure 3). The source of knowledge of contraceptives is given in the Supplemental File (File S3).

## Discussion

The present study found a high prevalence of unmet need for family planning (40.3%) among married women in rural Udupi district of coastal Karnataka. This prevalence is significantly higher than the National Family Health Survey-5 (NFHS-5)^6^ estimate of 7.1% for Udupi district. This discrepancy likely reflects the combined urban–rural sampling in NFHS-5 versus our rural-only focus, as unmet need is consistently higher in rural settings due to limited access and sociocultural barriers. Our finding underscores the persistence of gaps in family planning services in rural Karnataka despite overall progress at the district and state levels.

On further analysis, it was observed in our study that the unmet need for spacing was 13.5% while the unmet need for limiting was 26.9%. This starkly contrasts the NFHS-5 data at national and state levels, where the unmet need for family planning is lower (9.4% nationally and 6% for Karnataka).^6^ The predominance of limiting unmet need suggests that many women in our setting had already achieved their desired family size but were unable to access or adopt suitable long-acting or permanent contraceptive methods. This points towards programmatic shortcomings in counselling, service provision, and availability of such methods at the rural level.

The findings of the current study align with research conducted in Ethiopia by Gelegay et al.,^17^ who reported a comparable unmet need for family planning at 42.43% with higher unmet need for spacing (33.44%) than limiting (8.99%). However, the result of the present study is higher than those from rural areas of Kanchipuram (31%),^15^ Davangere district (16.7%),^18^ and Uttar Pradesh (21%)^19^ in India. This discrepancy may be attributed to sociodemographic factors, regional differences in healthcare access, education, and social norms around contraceptive usage patterns. Similarly, a study conducted by Anil et al.^20^ in Mysuru, India reported that the total unmet need for family planning was 29.01%, with 11.7% as the unmet need for limiting and 17.3% as the unmet need for spacing. Compared to these settings, our higher estimates highlight the need for targeted rural interventions in Udupi, particularly in strengthening access to limiting methods and addressing the barriers that prevent women from translating fertility intentions into contraceptive adoption.

The contraceptive prevalence rate (CPR) in our study was only 46% in our study, which is lower than the national (66.7%), state (69%) and Udupi district (72%) estimates reported in NFHS-5.^6^ This gap is consistent with other rural studies but contrasts with higher CPRs reported in urban and semi-urban areas such as Pune (63.8%)^21^and Coimbatore (69.5%).^22^ A study conducted by Sreedevi et al.^23^ among tribal population of Wayanad in Kerala state, India reported a CPR of 26.4% whereas Chelladurai et al.^24^ in their study among the women of reproductive age group in North-East India reported a CPR of 39.8% and Qazi et al.^25^ reported a CPR of 60% among women of reproductive age group in the state of Jammu and Kashmir, India. Fear of side effects, husbands working abroad, and reliance on traditional methods were cited as reasons for non-usage in our study, highlighting non-informational barriers. Other studies in urban and semi-urban settings have instead reported desire for more children as the leading reason for non-use (Taklikar et al.,^21^ Chaudhary et al.^26^), while apprehension about side effects was also widely observed (Dash et al.,^27^ Bhubaneswar). Together, these findings emphasise the need for method-specific counselling and community education to dispel misconceptions and promote modern methods.

Regarding awareness, 97.16% of participants were aware of at least one method of contraception. This shows that ASHA (Accredited Social Health Activists) workers, who are grassroot multi-purpose workers, have played a significant role in disseminating information. Although the awareness was high for contraceptives like male condoms, oral contraceptive pills, and tubectomy, it was alarmingly low for emergency contraceptives, with only 10.4% of participants knowing about it. This contrasts with NFHS-5 national data, where 57% of women were aware of emergency contraception, underscoring a significant knowledge gap. Despite nearly universal awareness, the uptake of contraception was limited, indicating a clear awareness–practice gap that merits further qualitative research to explore determinants of non-use.

Factors such as the age of the participants, place of delivery, number of desired children, and awareness of intrauterine devices were significantly associated with unmet needs for family planning. This aligns with findings from other studies conducted in various parts of India that have identified age, educational status, and the number of children as significant determinants of unmet needs for family planning.^28,29^ While the awareness about IUCD was high in our study, but the translation of this awareness into actual adoption remains limited. This knowledge-practice gap may be explained by several factors. First, despite knowing about IUCDs, women may harbour misconceptions about their safety and side effects, as evidenced by 13.2% of never users citing anxiety about side effects as a reason for non-usage of contraceptives. Secondly, provider biases might influence contraceptive method recommendations, particularly in private healthcare facilities where our study showed higher unmet need compared to government facilities.

The findings from this study emphasise the need for focussed interventions to bridge the gap between the high awareness and the low utilisation of contraceptives. Apprehension regarding the adverse consequences of the usage of contraceptives was the most cited reason. Individual counselling on family planning methods to dispel the misconceptions related to the adverse effects of contraceptive usage should be provided.

The strengths of the study include a good sample size and a pre-validated questionnaire with scope for evaluating multiple factors affecting unmet need of contraception such as knowledge and attitude towards family planning, use of contraception including emergency contraception usage, and information regarding sexually transmitted infections or reproductive tract infections.

The study included married women of reproductive age, ranging from 18 to 49 years. Pregnant women were excluded, as they did not have an immediate need for contraception. However, it is important to note that some of these women may have had an unmet need for family planning if their current pregnancies were unintended.

The study has certain limitations that should be considered while interpreting the findings. The prevalence of unmet needs was determined based on participants’ reproductive intentions and their current contraceptive use, which may be subject to recall bias and social desirability bias. Approximately 15% of the participants were between the ages of 41 and 49 years, falling into the perimenopausal and menopausal age range. Women who had reached menopause would not require contraception, but data on menopause status were not collected. Since data on menopausal status were not collected, the inclusion of women who no longer required contraception may have resulted in an overestimation of unmet need. In addition, a small proportion of women (3.9%) who had never used contraception cited fertility problems, but these women were not excluded from the computation, which may also have slightly influenced the prevalence estimates. Data on pregnant women and postpartum amenorrhoea were not collected in this study. Consequently, we could not account for these groups in the computation of unmet need for family planning. With respect to sampling, the study was restricted to rural areas of Udupi district, which limited the representativeness of the findings. Therefore, the results may not be generalisable to the urban populations or to other districts with different sociodemographic or cultural contexts. While the study provides useful insights into the local context, extrapolation to wider populations should be done cautiously and may require validation through larger, multi-centric studies.

## Conclusion

In rural Udupi, 40.3% of married women had an unmet need for family planning (13.5% for spacing; 26.9% for limiting), while contraceptive prevalence was 46%. Determinants included maternal age, place of delivery, fertility preferences (including desired number of female children), number of children desired at marriage, and awareness of IUCDs. Despite high awareness, fear of side effects, spousal migration, and reliance on traditional methods, limited uptake. These findings underscore the need for targeted interventions—counselling to address misconceptions, integration of family planning with delivery/postpartum services, and provider training to reduce biases—to bridge the awareness–practice gap. Future mixed-methods research should further explore context-specific barriers and inform tailored solutions.

## Recommendations from the present study

1. Strengthen Postpartum and Facility-based Counselling: Since place of delivery was significantly associated with unmet need, integrating structured family planning counselling into antenatal, delivery, and postnatal services, particularly in private facilities, could ensure timely information and method uptake.
2. Address the Awareness–Practice Gap: Although awareness of contraceptives was high, misconceptions and fear of side effects hindered uptake. Targeted community-based counselling, peer support groups, and use of trusted frontline health workers (e.g. ASHAs) can dispel myths and promote confidence in modern contraceptive methods.
3. Focus on Fertility Preferences and Gender Norms: The association between unmet need and fertility preferences, including the desired number of female children, highlights the role of sociocultural norms. Behaviour change communication campaigns should emphasise the benefits of smaller family sizes and promote gender equity in fertility preferences.
4. Target Younger Women and Newly Married Couples: Younger women and those deciding family size at marriage showed higher unmet need. Pre-marital and early marital counselling sessions, possibly through community outreach and adolescent health programmes, could help align fertility intentions with contraceptive use.
5. Expand Contraceptive Method Mix and Access: Improving availability and promotion of underutilised methods such as IUCDs and emergency contraception, with provider training to reduce bias and misinformation, will broaden choice and increase uptake.
6. Engage Men and Migrant Spouses: With spousal migration cited as a major reason for non-use, interventions should involve men through workplace-based and digital platforms, while promoting condom use and other methods suitable in such contexts.

## Supplemental Material

sj-docx-1-phj-10.1177_22799036251397747 – Supplemental material for Exploring the unmet needs of family planning: Insights from a cross-sectional study in a rural area of coastal Karnataka, India Supplemental material, sj-docx-1-phj-10.1177_22799036251397747 for Exploring the unmet needs of family planning: Insights from a cross-sectional study in a rural area of coastal Karnataka, India by Ratna Jay, Yash Alok, Manjula A., Ashwini Kumar and Sanjay Kini B. in Journal of Public Health Research

sj-docx-3-phj-10.1177_22799036251397747 – Supplemental material for Exploring the unmet needs of family planning: Insights from a cross-sectional study in a rural area of coastal Karnataka, India Supplemental material, sj-docx-3-phj-10.1177_22799036251397747 for Exploring the unmet needs of family planning: Insights from a cross-sectional study in a rural area of coastal Karnataka, India by Ratna Jay, Yash Alok, Manjula A., Ashwini Kumar and Sanjay Kini B. in Journal of Public Health Research

sj-pdf-2-phj-10.1177_22799036251397747 – Supplemental material for Exploring the unmet needs of family planning: Insights from a cross-sectional study in a rural area of coastal Karnataka, India Supplemental material, sj-pdf-2-phj-10.1177_22799036251397747 for Exploring the unmet needs of family planning: Insights from a cross-sectional study in a rural area of coastal Karnataka, India by Ratna Jay, Yash Alok, Manjula A., Ashwini Kumar and Sanjay Kini B. in Journal of Public Health Research
