# Supplementary material for: Exploring the unmet needs of family planning: Insights from a cross-sectional study in a rural area of coastal Karnataka, India
Source: J Public Health Res. 2026 Jan 20;15(1):22799036251397747. doi: 10.1177/22799036251397747 (PMC12819989; doi:10.1177/22799036251397747)
Supplement: sj-docx-1-phj-10.1177_22799036251397747 – Supplemental material for Exploring the unmet needs of family planning: Insights from a cross-sectional study in a rural area of coastal Karnataka, India [file sj-docx-1-phj-10.1177_22799036251397747.docx]

**Proportional allocation (PA) of study sample across the subcentres**

| Subcentre | No of eligible couples | Number of eligible couples selected by PA |
| --- | --- | --- |
| Subcentre A | 670 | 103 |
| Subcentre B | 715 | 110 |
| Subcentre C | 260 | 40 |
| Maatar and Padubettu | 450 | 69 |
| Kutyar | 397 | 61 |
| Belapu | 750 | 116 |
| Kalatoor | 420 | 65 |
| Total | 3662 | 564 |
