# Supplementary material for: Exploring the unmet needs of family planning: Insights from a cross-sectional study in a rural area of coastal Karnataka, India
Source: J Public Health Res. 2026 Jan 20;15(1):22799036251397747. doi: 10.1177/22799036251397747 (PMC12819989; doi:10.1177/22799036251397747)
Supplement: sj-docx-3-phj-10.1177_22799036251397747 – Supplemental material for Exploring the unmet needs of family planning: Insights from a cross-sectional study in a rural area of coastal Karnataka, India [file sj-docx-3-phj-10.1177_22799036251397747.docx]

Table 5 shows the association of awareness of contraceptives with the unmet need for family planning. The likelihood of unmet needs was greater among women who were aware of intrauterine contraceptive devices (IUCD) and injectables. This shows a divergence between the awareness about the methods of contraceptives and the practice of these methods.

**Table No.5:** **Association of awareness of contraceptives with the unmet need for family planning (n=549)**

| **Characteristic** | | **Unmet Need Present** | **Unmet Need Absent** | **p value** | **Unadjusted OR with 95% CI** |
| --- | --- | --- | --- | --- | --- |
|  |  | **Frequency (%)** | **Frequency (%)** |  |  |
| Male condoms | Yes | 223 (40.1) | 333 (59.9) | - | 1 |
|  | No | 2 (22.2) | 7 (77.8) | 0.291 | 0.427 (0.88-2.073) |
| Female condoms | Yes | 13 (38.2) | 21 (61.8) | - | 1 |
|  | No | 212(39.9) | 319 (60.1) | 0.845 | 1.074 (0.526-2.19) |
| OCP | Yes | 217 (40.5) | 319 (59.5) | - | 1 |
|  | No | 8 (27.6) | 21 (72.4) | 0.172 | 0.560 (0.244-1.287) |
| IUCD | Yes | 220(40.9) | 318 (59.1) | - | 1 |
|  | No | 5 (11.7) | 22 (15.3) | **0.027** | 0.329 (0.123-0.881) |
| Tubectomy | Yes | 220(39.9) | 331(60.1) | - | 1 |
|  | No | 5 (35.7) | 9 (64.3) | 0.751 | 0.836 (0.276-2.527) |
| Vasectomy | Yes | 142(39.2) | 220 (60.8) | - | 1 |
|  | No | 83 (40.9) | 120 (59.1) | 0.699 | 1.072 (0.755-1.521) |
| Injectables | Yes | 102(54) | 87 (46) | - | 1 |
|  | No | 140 (37.3) | 235 (62.7) | **0.001** | 0.415 (0.290-0.593) |

Table 6 denotes the association of source of knowledge with the unmet need for family planning. The odds of an unmet need for family planning were lower among participants who did not have family and friends as a source of information.

**Table No.6: Association of sources of knowledge with the unmet need for family planning (n=565)**

| **Characteristic** | | **Unmet Need Present** | **Unmet Need Absent** | **p value** | **Unadjusted OR with 95% CI** |
| --- | --- | --- | --- | --- | --- |
|  |  | **Frequency (%)** | **Frequency (%)** |  |  |
| Media | YES | 8 (25.8) | 23 (74.2) | - | 1 |
|  | NO | 217 (40.6) | 317 (59.4) | 0.107 | 1.968 (0.864-4.481) |
| Internet | YES | 47 (47.5) | 52 (52.5) | - | 1 |
|  | NO | 178 (38.2) | 288 (61.8) | 0.088 | 1.462 (0.945- 2.263) |
| Family and friends | YES | 134 (43.6) | 173 (56.4) | - | 1 |
|  | NO | 91 (35.3) | 167 (64.7) | **0.043** | 0.704 (0.5-0.989) |
| Health workers | YES | 199 (40.3) | 295 (59.7) | - | 1 |
|  | NO | 26 (36.6) | 45 (63.4) | 0.556 | 0.857(0.512-1.434 |

The age of the participant, the place of delivery of the last child, the ideal number of female children, the number of children the participant wanted at the time of marriage, and awareness of IUCD were significant factors associated with the unmet need for family planning. The odds of unmet needs were lower among participants under 30 years of age when compared to women who were older than 30 years. The odds of unmet needs among women who had their last delivery in a government hospital were lower when compared to women who delivered in a private hospital.
