# Supplementary material for: Exploring the unmet needs of family planning: Insights from a cross-sectional study in a rural area of coastal Karnataka, India
Source: J Public Health Res. 2026 Jan 20;15(1):22799036251397747. doi: 10.1177/22799036251397747 (PMC12819989; doi:10.1177/22799036251397747)
Supplement: sj-pdf-2-phj-10.1177_22799036251397747 – Supplemental material for Exploring the unmet needs of family planning: Insights from a cross-sectional study in a rural area of coastal Karnataka, India [file sj-pdf-2-phj-10.1177_22799036251397747.pdf]

Questionnaire

Sociodemographic information of the respondent

1	Participant no	
2	Age (in completed years)	
3	Address	
4	Religion	1.Hindu<br>2.Christian<br>3.Muslim<br>4.Others, specify
5	Education of the respondent	1. Illiterate<br>2. Primary<br>3. Secondary<br>4. PUC<br>5. Graduate and above
6	Occupation of the respondent	1.Student<br>2.Unemployed<br>3. Housewife<br>4.Unskilled worker<br>5.Semiskilled worker<br>6.Skilled worker<br>7.Proffessional
7	Education of the husband	1. Illiterate<br>2. Primary<br>3. Secondary<br>5. PUC<br>6. graduate and above
8	Occupation of the husband	1.Student<br>2.Unemployed<br>3.Unskilled worker<br>4.Semiskilled worker<br>5.Skilled worker<br>6.Proffessional
9	Family income (per month)	
10	Socioeconomic status based on Uday Pareek scale	

Marital and Obstetric history of the respondent

11	Age of the respondent at marriage (in completed years)	
12	Age of the respondent at birth of the first child	
13	Are you currently pregnant?	Yes No
14	Total No. of pregnancies	
15	Total No. of live births	
16	Total No. of abortions (if any)	
17	Total No. of children alive at present	
18	Total male children alive	
19	Total female children alive	
20	Place of delivery of the last child	i. Home<br>ii. Government health facility<br>iii. Private facility<br>Others (specify)
21	What was the spacing between the pregnancies?	

Knowledge and Attitude of the respondent toward family planning

22	In your opinion, what is the ideal age for a girl to get married?	i. 18-21<br>ii. 21-25 years<br>iii. 25-30 years<br>iv. >30years<br>v. Other specific answer
23	Which one is the ideal age for a woman to have first child?	i. Just after marriage<br>ii. 1-2 years after marriage<br>iii. >2 years after marriage<br>iv. Other specific answer
24	In your opinion what is the ideal number of children that a woman should have?	i. One<br>ii. Two<br>iii. More than two<br>Male: Female: No Sex differentiation
25	What is the ideal age space between two children?	i. One year<br>ii. One to two years<br>iii. Two to three years<br>iv. Three to five years<br>v. Five years or more<br>vi. Don't Know
26	When you started your family how many children did you and your husband want?	i. Never Thought of it<br>ii. One<br>iii. Two<br>iv. More than two

		Male: Female: No Sex Differentiation
27	After your marriage when did you and your husband want your first child?	i. Never Thought of it<br>ii. Just after marriage<br>iii. 1-2 years after marriage<br>iv. >2 years after marriage<br>v. Others (specify)
28	Do you want any more children?	Yes<br>No (skip to Qno 30)<br>Unsure (skip to Qno 30)
29	If yes, when and how many?	<24 months<br>>24 months
30	Did you ever discuss about these matters (the number of children and spacing) with your husband?	Yes<br><br>No (skip to 32)
31	If yes, then what was your husband's attitude?	
32	If no, why not?	
33	Did you discuss about these matters (the number of children and spacing) with anyone else?	
34	If yes, with whom?	i. Mother<br>ii. Mother-in-law<br>iii. Sister<br>iv. Sister-in-law<br>v. Friend<br>vi. Neighbour<br>vii. Other (specify)
35	Do you know about any services provided by the Government for family planning?	Yes No (skip to Qno 37)
36	If yes, specify.	
37	Is there any advantage of having a small family / family planning?	Yes No (skip to Q no 39)
38	If yes, what?	
39	Is there any disadvantage of having a small family / family planning?	Yes No (skip to Qno 41)
40	If yes, what?	
41	Did you get any education about family planning ?	Yes No
42	Source of education	a) Media<br>b) Internet<br>c)Family and friends<br>d)Health workers

		e) Others (specify)
43	Did anyone motivate you to use contraceptives?	Yes No
44	If yes, who?	I. Husband<br>ii. Mother<br>iii.Mother-in-law<br>iv.Sister<br>v.Sister-in-law<br>vi Friend<br>vii. Neighbour<br>viii. Health worker<br>ix.Others (specify)
45	Which family planning methods do you know about?	a) male condoms<br>b) female condoms<br>c) OCP<br>d) IUCD<br>e) tubectomy<br>f) vasectomy<br>g) others (specify)<br>h) don't know any method
46	From where did you come to know about these methods?<br>?	a) Media<br>b) Internet<br>c)Family and friends<br>d)Health workers<br>e)Others (Specify)
47	Have you heard/seen any message on family planning on radio/TV in the last six months?	Yes<br>No
48	Do you know of any incentive which is provided for family planning by the government?	Yes<br>No (skip to Qno 50)
49	If yes, specify	
50	Do you know any place where family planning services are provided free of cost?	Yes No (skip to Qno 52
51	If yes, which place?	a) Government hospital<br>b) Private hospital/clinic<br>c) Pharmacy<br>d) NGO / Trust hospitals<br>e) others (specify)
52	Did any health care provider visit your home after your marriage or pregnancy?	Yes No
53	How early can pregnancy be detected by urine pregnancy test?	

54	At present are you planning to get pregnant?	Yes No
55	Have you ever used any method of contraception?	Yes No
56	Are you currently using any of the contraceptive methods?	Yes No (skip to Qno 56)
57	If yes, What method are you using?	a) male condom<br>b) female condom<br>c) OCP<br>d) IUCD<br>e) tubectomy<br>f) vasectomy<br>g) others (specify)
58	If no, Why are you not using any contraception?	
59	Do you use it regularly?	Yes No
60	If yes, Duration of use	(in months/years)
61	If No, Why don't you use it regularly?	
62	Do you use any other method(s) when you are not using this method?	Yes No
63	If Yes, Which method(s)?	
64	Are you satisfied with the method you are currently using?	Yes No (skip to 64)
65	If yes, why?	
66	If no, why?	
67	Are you facing any problems regarding the usage of this method?	Yes No (skip to Qno 67)
68	If yes, what?	
69	Have you used any other contraceptive before using the current one in the last one year?	Yes No (skip to Qno 70)
70	If yes, Which method(s)?	
71	Why did you discontinue the method?	

For contraceptive former users

72	When was the last time you used a method?	i. Within 3 months<br>ii. More than 3-6 months ago<br>iii. More than 6 months -1 year ago<br>iv. More than 1 year ago
73	What was the method?	a) Barrier<br>b) OCP<br>c) IUCD<br>d) Others (specify)
74	Duration of use during last time	(in months)
75	Why did you stop using the method?	

For both contraceptive current and former users

76	Between you and your husband who decided to use this method?	i. Self<br>ii. Husband<br>iii. Both<br>iv. Others (Specify)
77	Did anyone oppose your decision of using contraceptives?	Yes No (skip to Qno 78)
78	If yes, who?	i. Husband<br>ii. Mother in law<br>iii. Father in law<br>iv. Sister in law<br>v. Whole Family<br>vi. Other (specify)
79	If yes, why?	
80	Did you suffer from any of the side-effects of this method?	Yes No
81	If yes, please specify	
82	Did you seek any treatment for these side-effects?	Yes No (skip to Qno 82)
83	If yes, what treatment and where?	
84	If no, then why not?	

85	From where did you procure this family planning method?	a) Health workers<br>b) Government hospital<br>c) Private hospital/clinic<br>d) Pharmacy<br>e) NGO<br>f) Others (specify)
86	Did you face any problem in procuring the contraceptives?	Yes No
87	If yes, specify	

For contraceptive non users

88	Why haven't you ever used any contraceptive method?	
89	Do you think you will use/continue to use any contraceptive method to delay or avoid getting pregnant at any time in the future?	Yes No
90	If no, why not?	

Emergency contraceptive usage and abortion related information

91	Do you know about emergency contraceptives?	Yes No
92	What methods of contraceptive pills are you aware of?	i. Emergency pill<br>ii. IUCD
93	In the last 12 months have you ever used emergency contraceptive?	Yes No
`94	If yes, how many times?	
95	Have you ever done requesting abortion?	Yes No
96	If yes, what was the reason for requesting abortion?	
